# Mesenchymal Stem Cells for Cryptoglandular Anal Fistula: Current State of Art

**DOI:** 10.3389/fsurg.2022.815504

**Published:** 2022-02-16

**Authors:** Chiara Eberspacher, Domenico Mascagni, Iulia Catalina Ferent, Enrico Coletta, Rossella Palma, Cristina Panetta, Anna Esposito, Stefano Arcieri, Stefano Pontone

**Affiliations:** Department of Surgical Sciences, Sapienza University of Rome, Rome, Italy

**Keywords:** cryptoglandular anal fistula, complication, recurrence, fecal incontinence, stem cells

## Abstract

Anal fistula is a common disease that needs surgical treatment to be resolved. Despite a variety of surgical options, the major problem is still to cure complex fistulas without any recurrence in the long-term follow-up but, at the same time, to avoid an impairment of continence. In recent years, one solution has been the application of mesenchymal stem cells derived from adipose tissue, especially in association with other treatments, such as the use of fibrin glue or the previous application of a seton. Their initial use in fistulas associated with Crohn's disease has shown encouraging results. In this non-systematic review our aim is to analyze the use in cryptoglandular fistulas: the rate of healing is not so high, and the number of studies is limited. Therefore, further randomized controlled trials are needed to establish their efficacy in the case of complex cryptoglandular anal fistulas and their possible complications.

## Introduction

Perianal sepsis with fistula and abscess is the most common anal disease, with an incidence of 2.32 cases per 10,000 people/year in Italy, ranging from 1.20 to 2.80 in Europe (1). The true incidence is probably underestimated: many patients can be treated with antibiotics before the diagnosis and the surgical treatment, with clinical regression of abscesses and symptoms; others can be drained in a physician's office (2). In most cases (95%), fistulas originate from a cryptoglandular infection with a preexisting abscess. Other causes for anal fistulas are inflammatory bowel disease (Crohn's disease [CD]), tuberculosis, trauma, irradiation, or other rare infections (3). Anal fistulas can be classified according to their location and complexity. Park's classification is one of the most common and relates fistula tracts with anorectal musculature, in particular internal and external sphincters (4). The American Gastroenterology Association added another classification, useful especially when associated with CD (5), differentiating simple and complex fistulas according to some parameters, for example, the number of orifices and the presence of anal strictures and proctitis. According to our consensus statement in Italy, simple anal fistulas are intersphincteric or low transsphincteric, with <30% of sphincter involved; complex fistulas are those associated with inflammatory disease, tumors, irradiation, incontinence, chronic diarrhea, and anterior localization in women (6).

Treatment of anal fistulas must be differentiated according to their type, complexity, and pathogenesis. Still, the final goal is always the same: to lead to complete healing and at the same time to preserve anal sphincter function and continence. For simple anal fistula treatment, seton and fistulotomy are recommended as the first choice, while the use of a novel technique seems to be unnecessary (6). In complex fistula treatment, there is a dichotomy between a “pure” surgical approach and new sphincter-preserving techniques. Fistulectomy with associated reconstruction with sphincteroplasty and anoplasty can be a solution with good perspectives in terms of healing, with a success rate of more than 90% (7, 8), but the postoperative worsening of continence, even if it occurs in a low percentage, is often judged intolerable by patients. The use of seton, considered to be the most ancient solution as first described by Hippocrates, is controversial for the same impairment of the anal sphincter, especially if we consider the use of a cutting seton. Endorectal advancement flap still has a high percentage of success with a mild effect on continence (6). According to different studies, ligation of the intersphincteric fistula tract (LIFT) and over-the-scope clip (OTSC) apposition (9) are two techniques centered on the closure of the internal orifice of the fistulas with a higher failure rate and heterogeneous outcomes (10–12). In recent years, the surgery technique has shifted to sphincter-preserving approaches. Anal fistula plug, video-assisted anal fistula treatment (VAAFT), and fistula tract laser closure (FiLac) are novel noninvasive strategies to treat both the internal orifice and fistula tract. Still, no clear percentages of healing are available from a literature review (13).

In recent years, novel techniques using staminal cells have changed the perspective: the approach is focused not only on the removal of the damaged tissue but also on a new regeneration of the tissue. The application of mesenchymal stem cells (MSCs) derived from adipose tissue in the treatment of anal fistula (adipose-derived stem cells [ASCs]) is the latest solution found to reach the ambitious target to heal perianal fistulas, without worsening the continence. Initial use in CD seems to be encouraging, while for cryptoglandular fistula the number of the study is limited. Our aim in this non-systematic review is to analyze results of application of ASCs in complex cryptoglandular fistula and the current state of art of the technique.

## Adipose-Derived Stem Cells and Rationale of the Application

MSCs are the most recognized multipotent cells. They can derive from a variety of tissues, including bone marrow and adipose tissue. These latest ASCs are adult stem cells that can be differentiated into mesoderm-derived tissue, such as adipose tissue, bone, cartilage, and muscle (14, 15). These cells also have intrinsic immunomodulatory properties, with the secretion of some anti-inflammatory molecules and paracrine signaling to nearby cells to maintain the local anti-inflammatory environment (15). The lack of substantial immunogenicity of MSCs allowed their use across human leukocyte antigen (HLA) barriers. They can repair damaged tissues and may achieve long-term healing of fistulas. The first application of ASCs in anal fistulas was in patients affected by CD (16), with encouraging results and rates of healing of 50%, in combination with anti-tumor necrosis factor (TNF) agents (17).

## Technique

ASCs are obtained by liposuction and processed to eliminate oily substances and blood cells, purifying them. Usually, under local anesthesia and general sedation, a small incision is performed and a cannula is introduced in the subcutaneous space. While the cannula is gently moved, the mild aspiration combined with the mechanical disruption of the fatty tissue allows obtaining the micro-fragmented adipose tissue, with a concentration of MSC 100 times higher than bone marrow aspiration (18). The site of lipo-aspiration is the abdominal region in the majority of cases (19). Techniques to purify the tissue have evolved over the years: earlier, solutions obtained from liposuction needed to be processed with collagenase, suspended, and centrifugated, with a subsequent time for the cells to be cultured (16). The number of transplanted cells ranged in different studies, and in some, the cell volume is proportional to the size of the fistula (19). In recent years, some devices have allowed the concentration of ASCs with mechanical micro-fragmentation and subsequent purification, without any other passages for procession or culture (20): the adipose tissue is washed, emulsified, and rinsed, and adipose cluster dimensions are gradually reduced to about 0.3–0.8 mm (21). The tissue, concentrated after decantation in some syringes to eliminate the excess saline solution, is then collected in 1-mL syringes (22) and injected. Anal fistula must be prepared before the injection with the curettage of the fistula tract and the closure of the internal opening (20, 23, 24). Injection is around the internal opening and all around the fistula tract from the internal to the external opening. Complications of the procedure are postoperative pain, fever, bleeding, and abdominal or perineal hematoma.

## Methods

**Figure 1:**
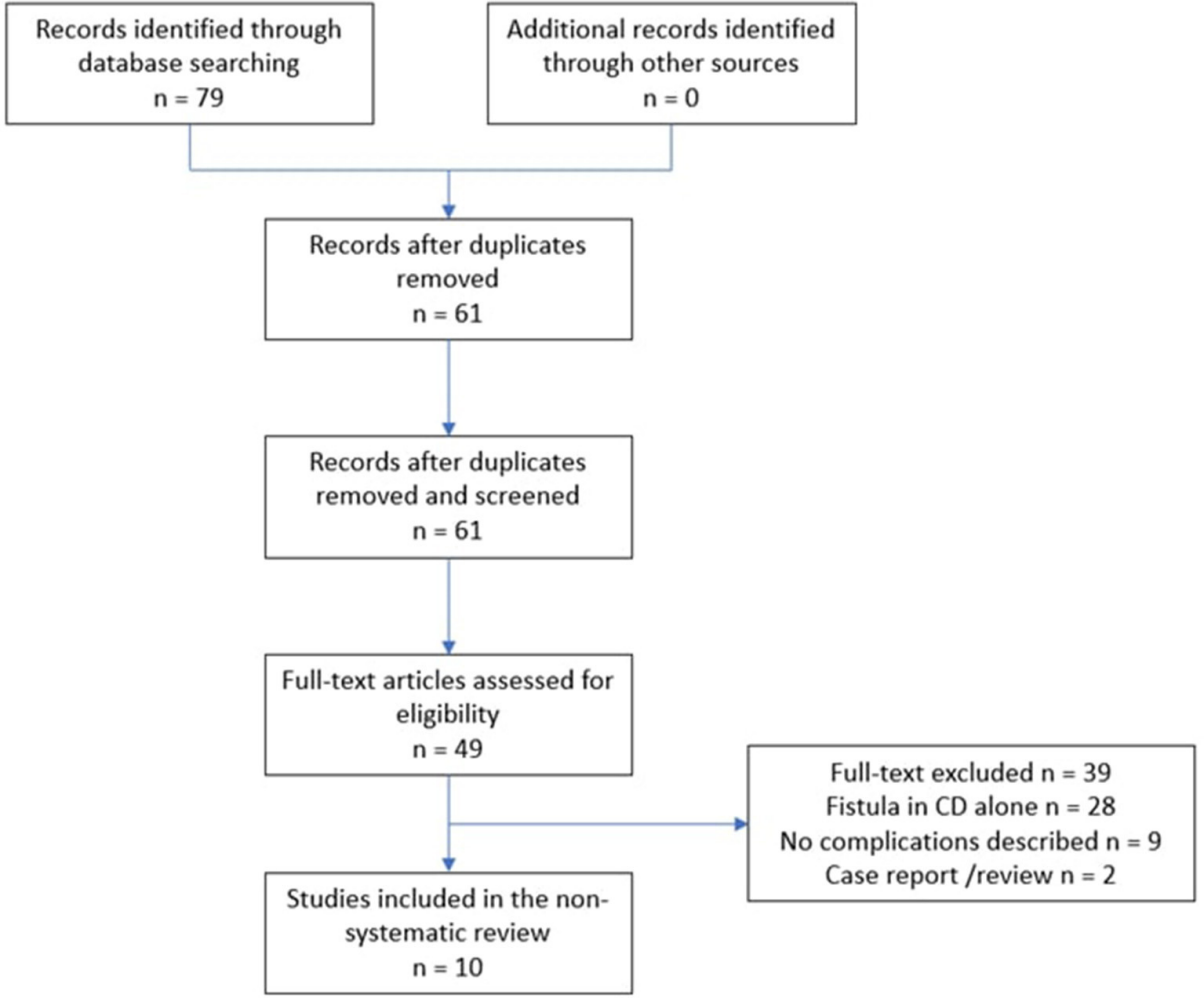
Flow chart of article selection process.

All studies that utilized ASCs to treat fistula-in-ano, from the period 2009 to 2021, were searched in PubMed, Medline, Scopus and Cochrane databases, and 79 articles were obtained. Keywords used for this search included “anal fistula”, “fistula in ano”, “adipose derived stem cells” and “mesenchymal stem cells”. Original studies, written in English were eligible for inclusion: we selected all randomized controlled studies, pilot studies and meta-analysis, excluding single-case studies, protocols, or reviews. Abstract were reviewed to identify full-text papers. We concentrated then on the application in cryptoglandular fistula alone to better understand the real efficacy in those cases, excluding the application in CD. We selected more recent publication, focused on the application of ASCs in cryptoglandular fistula: studies had to describe data on patient cohort, technique used and the association with other surgery, primary outcome described as clinical healing, complications and follow-up. Two reviewers (RP and CA) had to agree on inclusion of an article, if in total or substantial accordance with all the criteria of selection. They had all to be conducted according to PRISMA guidelines (Figure 1). In this way, we found 10 studies that analyzed the results of the application of ASCs in cryptoglandular anal fistulas. Patients were treated in association with other techniques (seton, fibrin glue, closure of the internal opening, anal plug) in almost every case of the application of ASCs. The total number of the patients considered in the review was 311. The rate of healing varied tremendously (from 20 to 73%). No major adverse events were reported with the procedure; fever and perianal abscess were common complications (Table 1).

**Table 1:** Mesenchymal stem cells for cryptoglandular anal fistula: clinical trials.

**References**	**Type of study**	**Type of fistula**	N. pts^*^	**Associated techniques**	**Rate of healing**	**Complications (Rate of Adverse Event)**
Garcia-Olmo et al. (25)	Phase II multicenter randomized controlled trial	Complex, both cryptoglandular and Crohn's	24 (15)	Fibrin glue	71%	Perianal abscess (5,7%)
Herreros et al. (26)	Multicenter randomized single-blind clinical trial	Cryptoglandular transsphincteric, suprasphincteric, intersphincteric	124 (183)	Fibrin glue or alone	40–42%	Proctalgia (43%), pain (13,7%), perianal abscess (13,1%), fever (9,3%), swelling (6,6%), pruritus (6,6%)
Borowski et al. (20)	Pilot study	Complex cryptoglandular	7	Closure of the internal opening with advancement flap	57.1%	Minor soiling (14,3%)
Naldini et al. (22)	Pilot study	Complex cryptoglandular transsphincteric	19	Fistula curettage and closure of the internal opening (also with flap)	73.7%	Abdominal hematoma (15,8%), perianal abscess (5,3%)
Garcia-Arranz et al. (27)	Randomized clinical trial	Complex cryptoglandular fistulas	23 (44)	Fibrin glue and closure of the internal opening	55.5%	Perianal abscess (4,3%)
Dozois et al. (28)	Phase I clinical trial	Single-tract transsphincteric	15	Anal plug	20%	Plug extrusion (6,7%), abdominal wall seroma (6,7%), perianal abscess (20%), perianal cellulitis (6,7%)
Topal et al. (24)	Single-center experience	Cryptoglandular transsphincteric, suprasphincteric, intersphincteric	10	Closure of the internal opening	70%	Hematoma (20%), perianal abscess (20%)
Zhang et al. (29)	Prospective case control study	Complex cryptoglandular	11 (total 24)	Previous seton, closure of the external opening	54.55%	Fever, perianal pain (27,7%)
Ascanelli et al. (30)	Randomized controlled Study	Complex cryptoglandular	58 (total 116)	Partial closure of the internal opening	63.8%	Abdominal wall hematoma (18,9%), Perianal abscess (1,72%), hemorroids complication (18,9%)
Maciel Gutiérrez et al. (31)	Nonrandomized controlled trial	Complex cryptoglandular	20	Previous seton, closure of the internal opening	69%	Perianal abscess (15%)

* *Number of patients treated with ASCs (total patients of the study)*.

## Results of ASCs in Cryptoglandular Anal Fistula

The first phase clinical trial investigated the use of ASCs derived from autologous fat in CD fistulas (16) in 2005. That was the first report of the application of MSCs in anal fistulas, even if only in five patients, showing a safe and feasible protocol. In 2009, the same group reported their result in a multicenter randomized controlled trial involving 35 patients with cryptoglandular fistulas and 14 patients with associated CD: the group of patients treated with the association of ASCs and fibrin glue presented a healing rate of 71 vs. 14% of the control group treated with fibrin glue alone (25). Fistula healing was defined as complete re-epithelialization of external openings here and in the other studies of the group. The FATT1 Trial further increased the number of patients, with 200 patients with cryptoglandular fistula, treated with ASCs, ASCs plus fibrin glue, and fibrin glue alone; in all patients, closure of the internal orifice of the fistula was performed as a phase of the injection: in the long-term results, fistula closure was reported in 57.1% (ASCs), 52.4% (ASCs + fibrin glue), and 7.3% (fibrin glue) (*p* = 0.13). The rate of healing doubled with the use of ASCs (26). In the evolution of the technique, new modalities to obtain ASCs without vigorous manipulation and the systematic curettage of the fistula tract and closure of the internal orifice guaranteed an encouraging healing rate after 2 years of application in the ASC group of 50% (27). The evolution of the technique to obtain micro-fragmented fat tissue, with a kit without the expansion of enzymatic treatment and the possibility to extract the tissue and inject the purified solution rich in ASCs in the same operative session, allows, in some cases, concluding the entire procedure in less than an hour, with the same quality of results: even if only in a small group, Naldini reports a healing rate of 73.7%, focusing on the importance of a rigorous closure of the internal orifice (22).

Recently, the use of ASCs was compared not only with fibrin glue but also with other surgical techniques, such as endorectal advancement flaps. Zhang reported an almost identical closure rate (54.55 vs. 53.85%), without any significant worsening in Wexner's score for incontinence (29). A phase I trial shows an association with a different technique for the closure of fistula tracts, the anal plug: 15 patients with trans-sphincteric cryptoglandular fistulas received a MSC-loaded fistula plug, with complete healing reported in three and “partial” in 7 (28). Another recent study on fistula surgery with or without injection of ASCs in complex anal fistulas reported that the healing rate was 63.8% in the experimental group compared with 15.5% in the group treated with surgery alone (*p* < 0.001), with a better postoperative course in terms of pain (30). A previous step with the seton drainage and closure of the internal opening seems to be the standardization in the technique, agreeing with a recent study in the literature (31). In recent metanalysis healing rate is similar (61,5%), in the group with cryptoglandular fistula treated with ASCs, but there is no a systematic description of complications (32).

Summarizing results of application of ASCs for treatment of cryptoglandular fistula, healing rate is similar or minor that described in CD. The description of healing is clinical, with some differences when Magnetic Resonance is used. In some trial we observed a success in more than of 70% of patients (22, 24, 27), but with fewer patients this rate of healing suddenly decreases (28, 29), also for the learning curve. Complications are frequent, even if major adverse events are rare and with an easy resolution: most common are perianal abscess especially at the beginning of the experience (27). The association with fibrin glue was constant in the first studies (26, 27), rare the use of other procedures like anal plug (28). In last year it seems to be essential for the technique to associate the closure of the internal orifice of the fistula tract (20, 22, 30, 31), with sometimes a previous application of a seton. It is not possible to evaluate the use of ASCs alone, without those described procedures, because considered as integral part of the technique.

## Future Perspectives and Conclusions

In the literature, the application of ASCs seems to be the preferred technique to obtain a high rate of success in healing complex fistulas in CD. Park also compared the use of medical therapy with anti-TNF agents and ASC injection and reported a faster healing of fistulas after seton placement in the group treated with ASCs (33). Despite some controversy, the administration of MSCs for perianal CD seems to be more effective than other treatments or medical therapy alone (17). Recent meta-analysis shows a high healing rate in randomized controlled trials (64% in the MSC arm vs. 37% in the control arm), without any major adverse events (34).

The use of ASCs in cryptoglandular fistulas is not as effective as in CD. A recent meta-analysis that included studies on both CD and cryptoglandular fistulas confirmed the efficacy of the procedure and safety, but with different results according to the origin of the fistula. MSC therapy is reported as effective for perianal CD healing (53.9%), with a statistically significant difference (*p* < 0:05), while healing in non-CD perianal fistulas was 49.5% (vs. 31.2% of the control group; *p* = 0.08), with no statistically significant differences (*p* > 0.05) (35).

Until now, ASCs have shown promising results in fistulas associated with CD, but not the same rate of success in cryptoglandular fistulas, especially when used alone. Their use is safe and effective, without impairment of the continence, but the association with other surgical techniques can increase their efficacy. Randomized controlled trials are still necessary to evaluate their widespread application, especially in cryptoglandular fistulas. The multipotent capacity of ASCs to differentiate provides new encouraging perspectives in proctology, especially to repair sphincteric damage (36). At the same time, the main concern about the application of MSCs is the possibility that they may promote the development of tumors, inducing neoplastic cell proliferation and neoangiogenesis (37), even if no case of tumor has been reported until now.

## Author Contributions

SP, DM, and CE: conceptualization. CE: writing–original draft. SP: writing–review and supervision. RP, CP, IF, AE, and EC: resources and investigation. SA: investigation and supervision. All authors contributed to the article and approved the submitted version.

## Conflict of Interest

The authors declare that the research was conducted in the absence of any commercial or financial relationships that could be construed as a potential conflict of interest.

## Publisher's Note

All claims expressed in this article are solely those of the authors and do not necessarily represent those of their affiliated organizations, or those of the publisher, the editors and the reviewers. Any product that may be evaluated in this article, or claim that may be made by its manufacturer, is not guaranteed or endorsed by the publisher.

## Abbreviations

CD: Crohn's disease
LIFT: ligation of the intersphincteric fistula tract
OTSC: over-the-scope clip
VAAFT: video-assisted anal fistula treatment
FiLac: fistula tract laser closure
MSCs: mesenchymal stem cells
ASCs: adipose-derived stem cells
HLA: human leukocyte antigen.
